# Estimating the lagged effect of price discounting: a time-series study on sugar sweetened beverage purchasing in a supermarket

**DOI:** 10.1186/s12889-022-13928-w

**Published:** 2022-08-06

**Authors:** Hiroshi Mamiya, Alexandra M. Schmidt, Erica E. M. Moodie, David L. Buckeridge

**Affiliations:** School of Global and Population Health, Department of Epidemiology, Biostatistics, and Occupational Health, McGill University, Suite 1200, 2001 McGill College Avenue, Montreal, QC H3A1G1 Canada

**Keywords:** Sugar sweetened beverages, Price discounting, Lagged effect

## Abstract

**Background:**

Price discount is an unregulated obesogenic environmental risk factor for the purchasing of unhealthy food, including Sugar Sweetened Beverages (SSB). Sales of price discounted food items are known to increase during the period of discounting. However, the presence and extent of the lagged effect of discounting, a sustained level of sales after discounting ends, is previously unaccounted for. We investigated the presence of the lagged effect of discounting on the sales of five SSB categories, which are soda, fruit juice, sport and energy drink, sugar-sweetened coffee and tea, and sugar-sweetened drinkable yogurt.

**Methods:**

We fitted distributed lag models to weekly volume-standardized sales and percent discounting generated by a supermarket in Montreal, Canada between January 2008 and December 2013, inclusive (*n* = 311 weeks).

**Results:**

While the sales of SSB increased during the period of discounting, there was no evidence of a prominent lagged effect of discounting in four of the five SSB; the exception was sports and energy drinks, where a posterior mean of 28,459 servings (95% credible interval: 2661 to 67,253) of excess sales can be attributed to the *lagged* effect in the target store during the 6 years study period.

**Conclusion:**

Our results indicate that studies that do not account for the lagged effect of promotions may not fully capture the effect of price discounting for some food categories.

**Supplementary Information:**

The online version contains supplementary material available at 10.1186/s12889-022-13928-w.

## Background

Sugar Sweetened Beverages (SSB) represent the largest source of dietary sugar in many nations [1] and are epidemiologically linked to obesity, overweight and nutrition-related chronic diseases [2]. Price discounting, the temporary reduction of price per unit food, is one of the most prevalent marketing tactics used by food retailers and manufacturers to increase sales [3, 4]. Price discounting is reported to have more consistent association with increased sales than other in-store promotions (e.g., display, flyer, and giveaway promotions) and media advertising [5]. Prevalence of price discounting is often reported to be disproportionately higher among highly processed ‘junk’ food including SSB [6], and people’s purchasing of SSB appears to be particularly susceptible to price discounting – more so than solid (non-beverage) food [7, 8]. Price discounting may lead to the overconsumption of the promoted food items [3, 9, 10], thus being a retail (in-store) environmental risk factor for food diets inconsistent with nutrition guidelines.

From an intervention perspective, price discounting is a highly unregulated and neglected environmental risk factor for unhealthy eating [11]. In addition, price discounting may be used as part of industry strategies to counter taxes on SSB, as suggested by the documented record of industry responses after tobacco taxation [12]. While a recent and the only study investigating industry responses to SSB taxations showed a decreased odds of price promotions after the tax is enacted [13], further research on potential changes in the influence and prevalence of price discounting is needed. The only regulatory initiative to date, delayed for enactment, is the UK government’s proposal for the mandatory restriction of volume-based discounting (e.g. reduced price for multi-buy) on food products high in fat, sugar and sodium [14]. Given the lack of interventions and natural experiment to study price discounting, evidence from observational studies characterizing the impact of discounting on population nutrition may provide motivative knowledge for governmental actions in other settings.

Several pioneering studies in public health nutrition found an association between discounting and increased sales of the promoted food items, mainly based on cross-sectional analyses that pooled purchasing and discounting records during the entire study period [5, 6, 15]. The findings are confirmed by the results from longitudinal studies controlling for time-varying confounders (e.g., season and other forms of time-varying marketing activities) including our previous work [3, 16, 17]. While the increase of sales during the period of discounting is consistently observed, time-lagged effect of discounting, or the association of discounting at current time with sales in the post-discounting time periods, has not received research attention.

A lagged effect of marketing exposure, including price discounting, can occur due to repeated purchasing of items after initial “try-out” purchasing triggered by marketing activities, a phenomena often termed purchase reinforcement [18]. Such lagged effects may be particularity strong (i.e., long lasting) if a product is introduced to a population that is unfamiliar or has not consumed similar products [3, 18]. These “novel” and fast growing products include sports and energy drinks and e-cigarettes that are diffusing into youth populations through non-traditional marketing channels such as social media websites and sport events [19–21]. Although the lagged effect of price discounting were investigated and confirmed by marketing researchers for some food categories [3, 22, 23], these findings do not readily apply to food groups of public health interest e.g., beverages may not be separated into diet (without artificially added sugar) and their non-diet (SSB) counterparts and often focus on sales for a small number of top-selling brands within a food groups of interest [23]. One study conducted by a marketing firm for Public Health England suggests the potential lack of such effect [24]. However, no longitudinal studies in public health nutrition specifically targeted the identification of lagged effects of price discounting (and cross-sectional studies are, by design, unable to estimate the temporal lag of an exposure effect). Lagged effect therefore remains as an unaddressed and overlooked factor in the association of price discounting (and other promotional activities, such as display and flyer promotions) with sales, potentially leading to previously unrecognized excess sales.

The objective of this study is to conduct a time-series analysis to assess the presence and magnitude of a lagged effect of discounting for five SSB categories based on weekly time-series of retail transaction data in a large supermarket in Montreal, Canada. The SSB categories of interest are 1) carbonated soft drinks (hereafter termed soda), 2) fruit drinks (less than 100% fruit beverages), 3) sports and energy drinks, 4) sugar-sweetened coffees and teas, and 5) sugar-sweetened drinkable, as opposed to spoonable, yogurt. These are non-alcoholic beverages containing artificially added sugars and not containing artificial sweeteners, thus excluding diets products. This is to our knowledge the first study to provide insights about the lagged effect of within-store obesogenic marketing activities.

## Methods

### Study design

This is a retrospective time-series study investigating the association between weekly discounting and sales of five SSB categories in a single supermarket located in Metropolitan Montreal, Canada. The study time period represents the period covered by our beverage transaction data, which is between January 2008 and December 2013, thus consisting of 311 weeks, or 6 years. The unit of analysis is weekly sales transactions for each beverage category. Note that this is not a longitudinal data analysis that uses measurements from multiple stores as seen in our previous studies [16, 17], i.e. these are not panel data. Rather, we performed a time-series (i.e., single store) analysis, which allowed us to explore time-lagged effects while accounting for temporal correlation of sales.

### Transaction data

The transaction records were generated by a large supermarket owned by a major Canadian retail chain (the identity of the chain is anonymized) and were purchased from a marketing firm, Nielsen [25].

The data consist of weekly sales quantity of individual beverage items, as uniquely defined by the Universal Product Code and item name, weekly price of sold items in Canadian cents, flyer promotion and retail display promotion (described below). We classified these items into the five non-alcoholic SSB categories based on product name of each beverage item and corresponding food category assigned by Nielsen. For example, soda items were categorised by the company as “carbonated soft drink”, but we manually excluded diet soda i.e., items with artificial sweeteners based on terms such as “diet”, “zero”, “non-sugar”.

### Outcome

The weekly sales quantities of each beverage item were standardized to the Food and Drug Administration’s single serving size of 240 ml for beverage (approximately 1 cup). The outcome variable was the aggregated sum of sales from items in each category in each week, where the category-specific average number of distinct items over the entire 6 year period in our store was 109 (soda), 152 (fruit drinks), 36 (sports and energy drinks), 22 (coffees and teas), and 29 (drinkable yogurts). The category-specific sales were natural log transformed to reduce skewness. We did not analyse the disaggregated, individual item-level association between sales and discounting, since such an analysis required us to account for across-item dependency of sales. Since the change of category-level sales is of primary relevance to population nutrition rather than the sales of individual food items or brands, our unit of analysis for both exposure, outcome and covariates was defined at the level of beverage category.

### Exposure

The exposure variable is category-specific discounting at each week. Specifically, it is a continuous variable calculated as the weighted average of weekly price discounting of individual items in each category, with weights representing each item’s market share (proportion of serving-standardized sales) within the category to which it belongs. Price discounting of an individual item is a continuous measure and was calculated as percent decrease of the serving-standardized price sold (net price) from the baseline (i.e., non-promoted) price [16, 26]. Detailed calculation of serving-standardized discounting for each item and subsequent aggregation to category is provided in Appendix S1 and Supplementary Fig. S1 in the Supplementary Information File.

### Statistical analysis: regression variables to capture lagged association of price discounting and SSB sales

A lagged association between time-varying outcome (log-transformed sales quantity) and exposure (discounting) is commonly captured by a distributed lag model, which is a regression model that contains multiple time-lagged values of an exposure. Regression coefficients for these time-lagged variables have functional constraints (i.e., the value of the coefficients is constrained to change smoothly over lag) as frequently seen in environmental time-series epidemiology and econometrics [27, 28]. One such constraint is the Koyck lag decay [29], which captures the monotonic decay of the effect of an exposure over time by two regression coefficients: *β* as the immediate effect (at lag zero) and λ as the lag coefficient that quantifies the decaying rate. The functional form of the Koyck decay is represented by a polynomial of form:

$$\beta {\lambda}^0+\beta {\lambda}^1+\beta {\lambda}^2+\beta {\lambda}^3+\dots +\beta {\lambda}^h,$$

where *h* indicates lag, and *βλ*^0^ = *β* is the immediate effect. An estimated value of the lag coefficient *λ* closer to 0 indicates the absence of a lag, while its value closer to 1 indicates a stronger lagged effect. The visual interpretation of the lagged effect represented by this polynomial function is provided in Supplementary Figs. S2 a and b (Appendix S2). We pre-specified the range of the estimated value of *λ* to be 0 < *λ* < 1 so that the effect of discounting decayed monotonically towards zero over the lag, capturing a diminishing effect.

### Statistical analysis: time-series regression model to incorporate Koyck lag model

The Koyck lag variables were added to a linear time-series regression, dynamic linear model [30, 31]. The details of the model, including the intercept and the lag coefficients, are provided in Appendix S3. We accounted for seasonal trends of sales by adding the sine- and cosine-transformed harmonic wave of a week variable as detailed in Appendix S3.

Covariates were weekly varying variables that are likely to temporally correlate with price discounting and sales. These included non-discounting promotion: weekly-varying display promotion and flyers, which often co-occur with price discounting (although not always) and are associated with higher sales [3]. Display promotion is temporarily placement of items into prominent location of stores such as store front. We calculated the value of these variables at the level of SSB category at each week by aggregating binary promotion status across items. Specifically, display promotion was coded as 1 if an item was temporarily placed at any one of prominent retail locations from the original shelf space, such as the end of aisle, entrance to store, or by the cashier. Flyer promotion was coded as 1 if an item was listed in flyer, and 0 otherwise. These item-level binary variables were aggregated to the category-level proportion as the weighted proportion of items promoted in each category at a given week, where the weights represented an item’s serving-standardized market share, as in the discounting variable. Additionally, an indicator variable for whether the week contained national and provincial statutory holidays was added. Other covariates were regular (baseline) price of each beverage categories, mean daytime temperature in each week, and the lagged value of sales itself (autoregressive of order 1).

We fitted a separate model for each of the five food categories independently under the Bayesian framework. We therefore specified prior distributions for regression parameters (Appendix S3). Interpretation of regression coefficients is based on point estimates (posterior mean or median) and uncertainty (95% Credible Interval [CI]) as summarized from the posterior distribution of the parameters approximated by Markov Chain Monte Carlo methods. We used the Stan software, which uses Hamiltonian Monte Carlo methods and accessed through the Rstan package in R software [32]. Model selection, specifically selecting a subset of variables from the covariates described above was guided by the value of the Watanabe-Akaike Information Criterion (WAIC) indicator of model fit [33]. As sales of many food categories are expected to have seasonal trends a priori, we did not perform any selection of the seasonal terms and thus they were retained in all models. A lower WAIC value indicates a better-fitting model. Codes are publicly available in an online repository [34].

As a sensitivity analysis, we tested an alternative shape of promotion decay by changing the constraint of the lag parameter *λ* from 0 < *λ* < 1 to −1 < *λ* < 0. The latter specification implies that, rather than assuming monotonic decay seen in Supplementary Figs. S2 a and b, we allowed the model to capture a so-called ‘post-promotion dip’ (Supplementary Figs. S3), a sharp reduction of sales below pre-discounting period immediately after discounting [3]. Theoretical explanations for the post-promotion dip are provided elsewhere [3, 35, 36].

The study was approved by the Institutional Review Board, Faculty of Medicine, McGill University (IRB approval#: A07-E45-16B), which did not require a written or verbal consent from human subjects, as the study used aggregated (store-level) secondary data. All methods followed the institutional guidelines and regulations.

## Results

### Descriptive analysis

The median sales quantity of the SSB categories in terms of standardized serving size across 311 weeks in the target store varied widely across the SSB categories, with soda and fruit drinks being the largest source of SSB sales (Table 1). However, these two categories, along with coffee and teas and potentially drinkable yogurt, exhibited a mildly decreasing trend during the study period (Supplementary Fig. S4) relative to that of sports and energy drink, consistent with the trends between 2004 and 2015 in Canada [37]. The sales of sports and energy drinks exhibited strong seasonal (cyclic) patterns in this store but did not show the prominent increase in Canada and worldwide in the same time period and reported elsewhere [37, 38]. Discounting of soda, fruit drinks, energy and sports drinks, and sweetened coffees and teas appears to have modestly increased trends over time (Supplementary Fig. S5) relative to that of drinkable yogurt. Average percent discounting over the study period was highest for soda and lowest for yogurt (Table 2). Mean and median regular (non-discounting) price per serving were the highest for sweetened drinkable yogurt followed by sports and energy drinks, and the remaining 3 categories had far lower baseline prices (Table 3). The store neighborhood, as defined by Forward Sortation Area (first 3 digits of Canadian postal codes) in which the store was located had comparable census characteristics to the larger Canadian Census Metropolitan Area of Montreal consisting of 196 Forward Sortation Areas (Table 4), as measured by the 2011 Canadian National Household Survey) [39]. However, the store neighbourhood had a notably larger proportion of recent immigrants.

**Table 1:** Summary of weekly standardized sales quantities of SSBs in the target store between 2008 and 2013, in non-log scale of serving quantity

SSB category	Mean	Median	IQR
Soda	20,330.7	19,062.5	14,057.4, 25,351.9
Fruit drinks	14,237.9	14,303.5	10,887.3, 17,092.1
Sports and energy drinks	1400.4	1178.4	869.5, 1763.2
Sweetened coffees and teas	1514.5	1397.0	1062, 1825.3
Sweetened drinkable yogurt	1368.8	1226.7	991.4, 1594.5

Abbreviations: *IQR* Interquartile Range

Unit is servings (240mililiters per serving).

**Table 2:** Summary of weekly percent discounting per serving of SSBs in the target store between 2008 and 2013

SSB category	Mean	Median	IQR
Soda	18.0	17.3	12.6, 24.3
Fruit drinks	11.8	9.8	5.5, 16.3
Sports and energy drinks	8.4	8.2	1.0, 12.5
Sweetened coffees and teas	15.5	13.8	7.6, 21
Sweetened drinkable yogurt	6.3	4.4	2.0, 9.5

Abbreviations: *IQR* Interquartile Range

**Table 3:** Summary of weekly baseline (non-discount) price per serving of SSBs in the target store between 2008 and 2013

SSB category	Mean	Median	IQR
Soda	56.8	57.8	52.0, 61.0
Fruit drinks	48.2	48.2	46.7, 49.9
Sports and energy drinks	130.1	123.7	107.2, 146.7
Sweetened coffees and teas	56.1	57.7	49.3, 64.5
Sweetened drinkable yogurt	161.6	161.0	158, 164.9

Price is based on Canadian cents per 240 ml of beverages.

**Table 4:** Comparison of census characteristics between store neighborhood measured at the level of forward sortation area and those of all forward sortation areas in the Census Metropolitan Montreal, 2011 Canadian National Household Survey

Areal census characteristics	Store neighborhood	Median (Metropolitan Montreal)	IQR (Metropolitan Montreal)
Median household income in Canadian dollar	68,627.00	72,926.00	(46,411.65, 105,567.45)
Proportion of residents without post-secondary diploma or certificate ^a^	0.67	0.70	(0.56, 0.88)
Dwelling density per square kilometer	1195.03	1078.36	(75.95, 5411.71)
Proportion of household with children	0.47	0.46	(0.27, 0.58)
Proportion of recent immigrants ^b^	0.41	0.22	(0.03, 0.51)

Abbreviation: *IQR* Interquartile Range

Median and IQR are calculated from census variables in all forward sortation areas in the Census Metropolitan Montreal.

^a^ Among residents 25 years or older

^b^ Landed to Canada within 10 years

### Time-series regressions

The summary of the time-varying intercept for each of the SSB categories shows its temporal path capturing the local fluctuations and overall declining trends of the SSB sales, as seen in Supplementary Fig. S6 a-e. Inspection of autocorrelation plots of residuals suggests little serial correlation (Supplementary Fig. S7). The final set of covariates included the holiday indicator for all SSB categories, in addition to display promotion and regular (non-discount) price for some SSB categories (Supplementary Table S1). As noted above, all models included sine- and cosine-transformed harmonic wave of a week as covariates with no selection performed on these time-related (seasonal) variables.

### Immediate effect of discounting

**Fig. 1:**
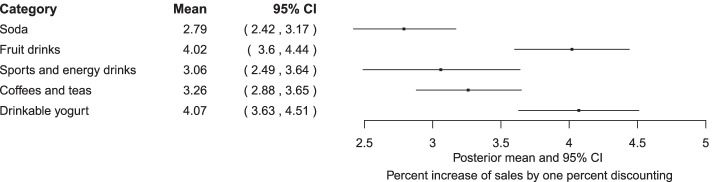
The estimated immediate effect β for price discounting on the sales of five sugar-sweetened beverage categories. The value represents the percent increase of non-log sales upon 1 % discounting for each SSB category, as calculated by multiplying the posterior summary of *β* by 100

The estimated coefficient *β* indicating the change of sales during the time of discounting is shown in Fig. 1. The values represent the change in natural log-transformed serving-standardized sales quantity by 1 % increase of price discounting during the period of discounting, which is equivalent to the *percent* change of non-log sales when multiplied by 100. This immediate effect was highest for drinkable sweetened yogurt and lowest for soda (Fig. 1). In other words, sweetened drinkable yogurt category is subject to the highest “deal-proneness” among the five SSB categories in this store. Soda beverages show the weakest immediate effects.

### Lagged effect of discounting

**Fig. 2:**
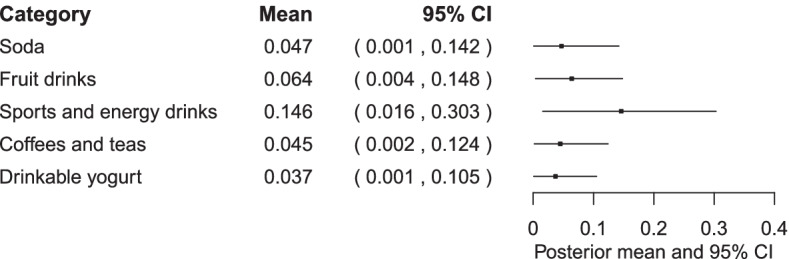
Posterior summary of the estimated lag coefficient, *λ,* for price discounting on the sales of five sugar-sweetened beverage categories. The variable *λ* represents a unitless quantity, whose value ranges from 0 to 1, with 0 representing the absence of lag

**Fig. 3:**
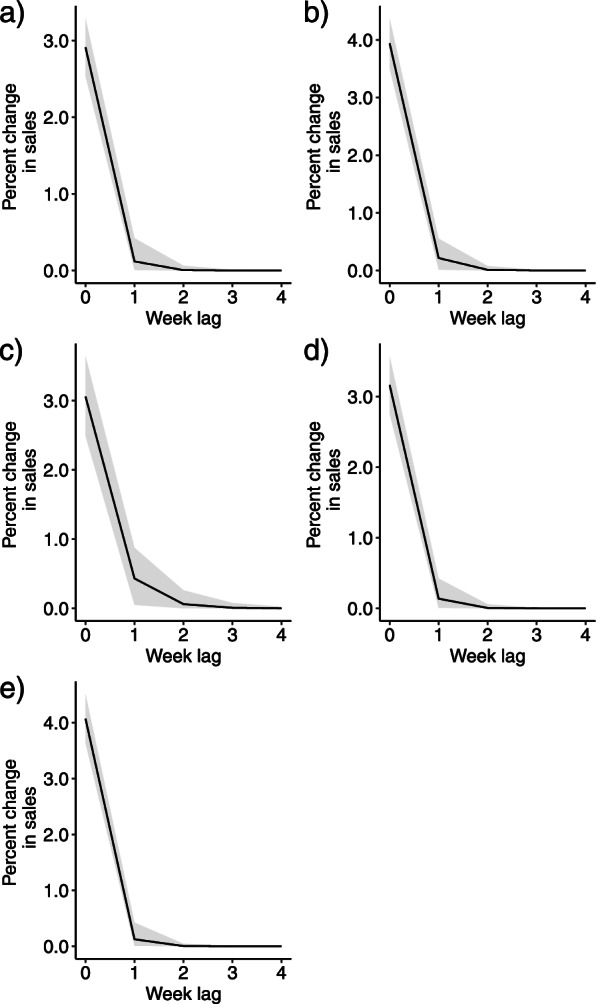
Impulse response function showing the lagged effect of price discounting on the sales of a) soda, b) fruit drinks, c) sports and energy drinks, d) sweetened coffees and teas, and e) sweetened drinkable yogurts, with 95% Credible Interval indicated by the gray shaded area. The value at *x* = 0 represents the immediate effect represented by the posterior median of *β*, the percent change of sales during the period of 1 % discounting

The extent of lagged effect (the coefficient λ) for each food category is provided in Fig. 2. The estimated value of *λ* is close to zero for all SSB categories but somewhat larger for sports and energy drinks. The visual interpretation of the lagged effects in the form of the above mentioned Koyck polynomial function for each SSB category (Fig. 3) indicate that the percent increase of sales relative to baseline (pre-discounting period) immediately drop to almost zero at lag 1. These shapes suggest a diminishing effect immediately after the period of discounting (i.e., lag 0), except for a weak and therefore short-term lagged effect for sports and energy drinks.

We also quantified the absolute total excess sales of each SSB categories attributable to the lagged effect alone over the 6 years of observation of discounting in our store (Supplementary Table S2). This is the posterior distribution of the difference between exponentiated fitted sales generated by the model containing the lag parameter λ and the exponentiated fitted sales generated by the model with the immediate discounting effect alone (i.e., setting λ = 0). For sports and energy drinks, these excess sales due to lagged effects of discounting are summarized by a posterior mean of 28,459 (median = 26,345, with 95% CI 2661 to 67,253) servings, which is approximately 21% of the sales quantities attributable to the total (immediate plus lagged effect of price discounting combined, posterior mean = 131,606, median = 130,446, 95%CI = 96,155 to 173,625).

A sensitivity analysis to inspect the presence of the post-promotion dip effect using alternative constraints of the lag coefficient (−1 < λ < 0) showed inferior model fit to the original specification (0 < λ < 1) which modelled a monotonic decay of discounting effect. We also performed additional analyses applied to sports and energy drink categories separately and to diet soda (soda containing artificial sweetener rather than sugar) products (Supplementary Figs. S8, S9 and S10). The results for sports and energy drink are similar to those from the main analysis grouping the two categories together, showing a conclusive lagged effect but wider 95%CI. As in its sugar-sweetened counterpart, diet soda did not show evidence of a lagged effect.

## Discussion

We investigated time-lagged effect of price discounting for five SSB categories for a supermarket located in Metropolitan Montreal, Canada. The results indicate that the association between discounting and sales of sports and energy drinks persisted even after discounting ended. To the best of our knowledge, the extant public health research estimating the association of price discounting and sales has evaluated only the immediate effect, thus potentially not capturing the total (immediate and lag) effect price discounting on the sales of some food categories.

There is an increasing number of studies investigating within-store food promotions as a modifiable obesogenic environmental drivers of (un)healthy food selection and nutrition disparities [5], and price discounting is likely to have the most influential impact on food purchasing [4, 23]. Similar to the exposure to environmental stressors (e.g., pollution, heat wave), lagged effect of marketing exposure in longitudinal and time-series analysis should be considered to be one of potential sources of bias, as seen in this study and literature in marketing science [3], as well as recent research investigating the impact of media advertising on population nutrition [40].

The lagged effect on the SSB category of sports and energy drinks may have occurred due to repeated trials induced by discounting among peoples who are previously unexposed to the consumption of these rapidly expanding SSB beverages, thus inducing purchase reinforcement. Sales and consumption of these beverages, in particular energy drinks, exhibited a steady and global growth during the study period [17], mainly propelled by aggressive and ubiquitous marketing activities within and outside retail settings, including sponsoring of sports and youth events [19, 41, 42]. While the percent increase of sales due to the lagged effect appears modest relative to the immediate effect, the absolute quantity of sports and energy drinks attributable to the lagged effect is concerning. Aside from their sugar contents, a single serving of energy drinks often reaches the recommended daily dose of caffeine intake among youth [43] and associated with caffeine-related acute health outcomes including neurological, psychological and often fatal cardiovascular events [42, 44].

Possible reasons for the absence of discounting carryover effect in the other SSB categories include rationale planning of shopping activities i.e., not buying items until next promotions [3]. This forward-looking planning may be relevant for categories that are discounted heavily, namely soda, fruit drinks and coffees and teas as seen in the descriptive analysis. As well, the lower baseline prices of these three categories may have further diminished the lagged effects discounting. It is also possible that the lagged effect is masked by the aggregated measure of sales and discounting by SSB categories in this study. In other words, individual food items within categories may exhibit a lagged effect, but the increased sales due to such effects maybe an expense of reduced sales on competing items within the same category – often termed as “cannibalization” due to people’s switching of food items within a category [3]. Thus, the overall category sales might not have increased at post-discounting period. This explanation also applies to the results of the sensitivity analysis: the lack of post-promotion sales dip frequently observed in the disaggregated brand-level analysis [35, 36].

While it is reassuring that the lagged effect is absent for the SSB categories such as soda in the store investigated, the presence of such effect for the sales of sports and energy drinks implies potentially unaccounted sales due to lagged effects in previous studies targeting these beverages, including our previous study [17]. Therefore, performing lag analysis in studies investigating the influence of food marketing exposure is warranted. We remark that, while the analytical approach provided in this study is a flexible form of distributed lag model (no need to specify the lag length a priori), there are alternative and readily implementable regression models to capture lagged effects built upon the past two decades of lag analysis on exposure-outcome associations in environmental epidemiology [27, 45–47]. Although our study focused on capturing linear exposure-outcome lagged associations between discounting and sales, existing lag models, including our transfer function models, can readily incorporate non-linear exposure-outcome associations as well [29, 47, 48]. These methods are accessible as existing software libraries (typically implemented within a frequentist framework) obviating the need for complex statistical programming [27, 49, 50]. Our study also highlights the need for consumer behavior (individual shopper-level) research investigating behavioral explanations for time-lagged purchasing in response to price discounting and potentially other forms of promotions, which are important food environmental exposures and may also modify the effectiveness of policy interventions, such as beverage taxation.

Our findings should be interpreted with several limitations in mind. First, while one of the key contributions of this study is to introduce an exposure lag modeling approach applicable to other populations, the data in this study are not recent (2008–2013). Given that the sales of energy drink are forecasted to grow further [51], the study motivates further investigation to confirm lagged effects on more recent sales and promotion data. As well, our findings are based on shopping patterns in a single supermarket. Population-level influence of discounting across varying socio-economic status at the shopper- or store neighborhood-level needs to be estimated based on a regionally representative sample of stores or people. This would require panel data, which in turn would bring significant increases in the computational complexity, requiring hierarchical analyses of lagged models with spatial correlation across geographical locations of stores, which remains our future research. As in any observational study, we note the potential for unmeasured confounders of price discounting, such as media advertising or a community or school-based health promotion program that took place near the target store. We also note that potentially important individual product-level information, such as the size of products (e.g., 2.0 L bottle vs. 350 ml can) and flavour were not accounted for, as they are masked by the aggregation of items at the level of category. Finally, it is possible that potential switching of SSB purchasing in nearby stores led to measurement error in our store, although the proportion of individuals prone to switch shopping venues appears to be relatively small (10–15%) and this pattern of store-substitution more typically occurs for high-cost items such as coffee and beer [52, 53].

Future research should investigate the lagged effect of other forms of sales promotions, including couponing, volume discount, display and flyer promotions, which independently and jointly influence selections of energy-dense and nutritionally poor food items [3, 5].

## Conclusions

Overall, our results provide insights into the lagged effect of price discounting on unhealthy beverage purchasing that should be further investigated by other observational studies, as such effect may represent a previously overlooked source of bias in the association of sales and within-store food marketing activities, which is recognized as a potentially important but largely unregulated component of obesogenic food environment.

## Supplementary Information

**Additional file 1.**

## Data Availability

Scanner transaction data from retail food outlet used in this study are collected in many nations by the Nielsen company (https://www.nielsen.com/ca/en/solutions/measurement/retail-measurement/). The data are available through commercial agreement with the company or through affiliated academic institutions that maintain licence to access to these data for research use.

## Abbreviations

SSB: Sugar Sweetened Beverages
CI: Credible Interval
