# Supplementary material for: Estimating the lagged effect of price discounting: a time-series study on sugar sweetened beverage purchasing in a supermarket
Source: BMC Public Health. 2022 Aug 6;22:1502. doi: 10.1186/s12889-022-13928-w (PMC9356513; doi:10.1186/s12889-022-13928-w)
Supplement: Supplementary file 1 — Additional file 1. [file 12889_2022_13928_MOESM1_ESM.docx]

**Supplementary Information File**

**Title:** Estimating the lagged effect of price discounting: a time-series study using transaction data of sugar sweetened beverages.

**Authors:**

Hiroshi Mamiya, Alexandra M. Schmidt, Erica EM. Moodie, David L. Buckeridge

Contents

[Appendix S1. Detailed Description of Calculation of Category-Level Discounting 2](#_Toc109245212)

[Supplementary Figure S1. 3](#_Toc109245213)

[Appendix S2. Shapes of promotional lag 5](#_Toc109245214)

[Supplementary Figure S2 5](#_Toc109245215)

[Appendix S3. Statistical analysis: time-series regression to incorporate the lagged effect of discounting, covariates, and intercept 6](#_Toc109245216)

[Supplementary Figure S3. 9](#_Toc109245217)

[Supplementary Figure S4 10](#_Toc109245218)

[Supplementary Figure S5 11](#_Toc109245219)

[Supplementary Figure S6 12](#_Toc109245220)

[Supplementary Figure S7 13](#_Toc109245221)

[Supplementary Table S1 14](#_Toc109245222)

[Supplementary Table S2 14](#_Toc109245223)

[Supplementary Figure S9. 15](#_Toc109245224)

[Supplementary Figure S10. 16](#_Toc109245225)

[References for Supplementary Information File 16](#_Toc109245226)

# Appendix S1. Detailed Description of Calculation of Category-Level Discounting

As in previous studies, we used a conventional marketing approach to calculate the category-level discounting of Sugar Sweetened Beverages (SSB) for each store-week from store-level scanner data (Raju, 1992).

The terms used in the analysis are defined as:

1) *Item* refers to a unique beverage product as identified by Universal Product Code in transaction data. A large supermarket often carries more than 1000 distinct SSB items.

2) *Net price* indicates the price of a SSB item after discounting, standardized to a single serving size (240ml).

3) *Regular price* is the baseline (reference) price of a single serving without any discounting, and

4) *Item* *discounting* indicates the difference between the net and regular price, thus the extent or depth of temporary price reduction per serving. Note that net and regular price are in Canadian Cents.

Because the data provide only the weekly net price after discounting for each item but not the regular price nor discounting directly, we calculated the regular price for each item, and then we computed discounting as the difference between the calculated regular price and the observed net price. We then computed the category-level discounting as the weighted mean of item discounting across items in the same week, with the weights representing the market share of each item.

For simplicity, we focus on the calculation of discounting for the soda category as an example. However, the calculations below were applied to each of the five SSB categories. Let $i$ represent an item of soda sold at week $j$. We denote the net price of item $i$ at week $j$ as $P_{ij}$. We examined the price history of each item. The regular price of soda item $R_{ij}$ is identified as the highest price in a 3-month window, e.g., a moving maximum net price of item $i$ in a 3-month window. Supplementary Figure S4-1 below illustrates a series of weekly net price of one of popular beverage items and its regular price.


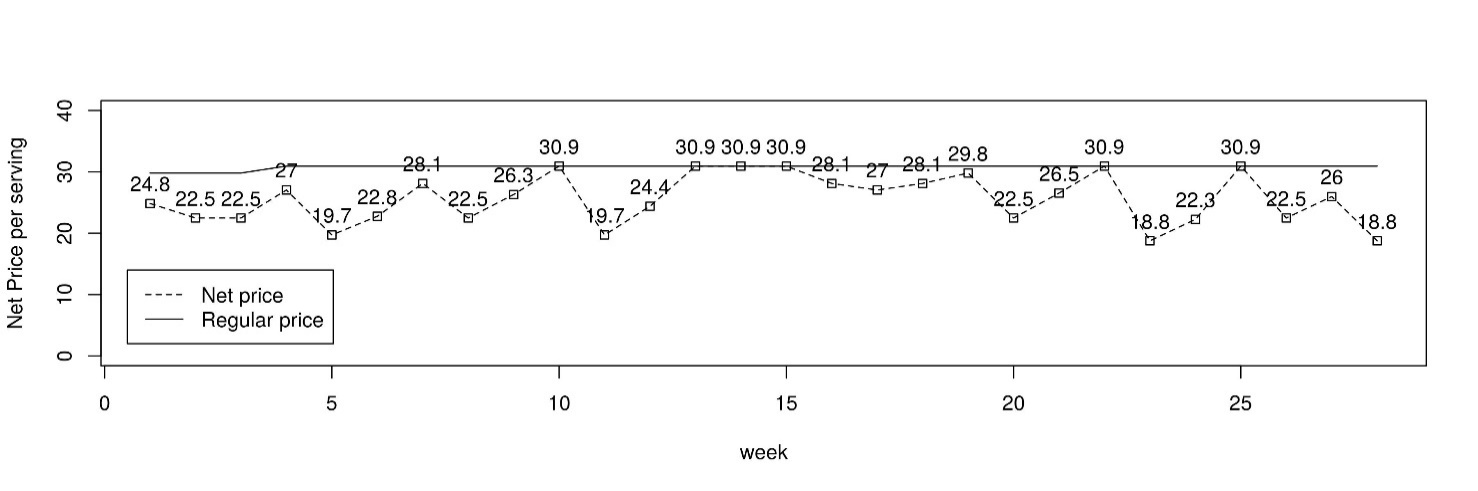


Supplementary Figure S1. Weekly Net Price and Regular Price of a Single Soda Item in Cents.

Item discounting, $D_{ij}$ , is defined as a percent price decrease from regular price as shown below;

$D_{ij}$= ${(R}_{ij}$ – $P_{ij})$ / $R_{ij} \times100$

Supplementary Figure S1 indicates that there is no item discounting (zero percent item discounting) in weeks 10, 13-15, 23, and 25, so the net price is the same as the regular price for these weeks. In other weeks, there is discounting since the net price is less than the regular price. In week 11, for example, the discounting is computed as: (30.9 – 19.7) / 30.9 x 100 = 36.25 percent.

Finally, we aggregated the individual item discounting into an overall measure for the discounting in the each of five SSB categories. Suppose there are 300 soda items sold in week $j$. Our main exposure of interest, the category-specific week discounting of all soda items, $X_{j}$,is defined as

$X_{j}=\sum_{i=1}^{300} D_{ij}W_{i}$ *,*

where $W_{i}$ represents a normalized (sum to one across all items) weight calculated from the annual market share of each soda items within the soda category. Therefore, the value of the weights is large and stable over time for soda items of major brands.

# Appendix S2. Shapes of promotional lag


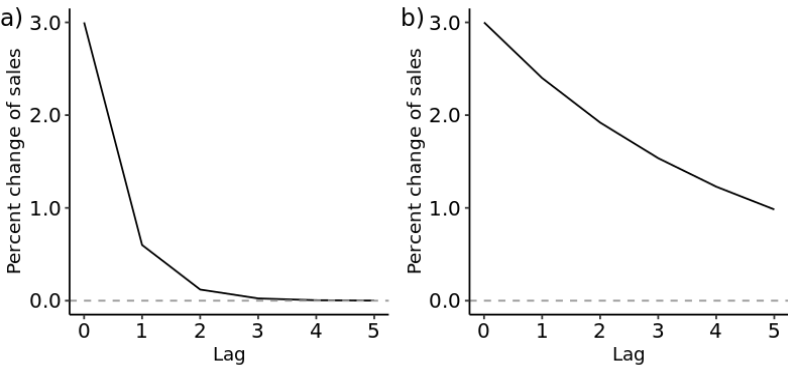


Supplementary Figure S2: Hypothetical shapes of the lagged effect of price discounting as a) a monotonic decay returning to the original (baseline, pre-promotion) level of sales with a weak estimated lag effect ($\lambda=0.2)$, and b) a more persistent lag ($\lambda=0.8)$ such that sales have not decayed to pre-discounting levels after 5 weeks.

The functions seen in panels a) and b) of Figure S2 are generated when the value of $\lambda$ is constrained to be between 0 and 1 by an analyst.

Discounting in this plot represents a ‘shock’ exposure, that is, its value is increased by one unit (i.e., one percent discounting) at the week where *x* = 0 (promotion period), then set to zero, or returning to the baseline, pre-discounting, price in the following weeks.

The y-axis represents the effect of one percent discounting on sales at each week. The value at *x* = 0 represents the immediate effect, which is the change of sales during the week of discounting, as represented by the value of $\beta$.

# Appendix S3. Statistical analysis: time-series regression to incorporate the lagged effect of discounting, covariates, and intercept

*Transfer function*

First, we specify the Koyck lag specification discussed in the main text. The monotonic decay of discounting at time *t* is summarized by a structural variable $E_{t}$ as:

$E_{t}= \lambda^{0}\beta x_{t} + \lambda^{1}\beta x_{t-1} + \lambda^{2}\beta x_{t-2} +\ldots+ \lambda^{h}\beta x_{t-h}$,

where $x_{t}$ represents price discounting at time *t*, $\beta$ is the coefficient for the immediate effect of discounting, $\lambda$ is the decay parameter as described in the main document, and *h* indicates lag length. The equation above allows the effect of discounting to geometrically decay over lag. Using transfer function, the same equation can be recursively summarized as

$E_{t}= \lambda E_{t-1} + \beta x_{t}$,

which indicates that the structural variable $E_{t}$ captures immediate effect of $x_{t}$ and its cumulative effect up to time *t* through lagged variable $E_{t-1}$ (1). Ravines et al. provide a review of distributed lag models and transfer functions (2).

*Time-series model*

We added the structural variable $E_{t}$to a dynamic liner model, a time-series linear regression model that naturally incorporates time-varying parameters, crucially $E_{t}$ above, to smoothly change over time under structural formulation (1,3). In our analysis, the model is specified as:

$$y_{t}=\alpha_{t}+{Season}_{t}+\delta C_{t}+E_{t}+\epsilon_{t}$$

$E_{t}= \lambda E_{t-1} + \beta x_{t}$ ,

where $E_{t}$ is the structural variable capturing time-varying influence of discounting combining the immediate and lagged effects up to time *t* as descried in the main text. The outcome is the natural log-transformed sales outcome of each SSB category denoted as $y_{t}$, with the error term $\epsilon_{t}$, which is assumed to follow a zero mean normal distribution and is independent across time.

The intercept $\alpha_{t}$ is a ‘dynamic’, or time-varying intercept, which captures long-term trends and temporal fluctuations of sales. This is a series of $\alpha$s (one for each week, and thus denoted as $\alpha_{t}$), interpreted as baseline sales at time *t* and is determined by its previous value, $\alpha_{t-1}$and random noise, hence this process is called a random walk. Further details in the “local level model” specification are available in references (1,3).

The term $C_{t}$ represents a vector of covariates described in the main document, the corresponding vector of time-fixed coefficients is denoted as $\delta$. The term ${Season}_{t}$ consists of nonlinear regressors as defined by sine and cosine functions fitted to the harmonic wave, with its regression coefficients controlling amplitude of the seasonal wave. Its periodicity is $2\pi t\omega$, where $t=1,2\ldots, 311$ as the indicator of week, $\omega$ as 52.2 representing the cycle (number of weeks in a year) and $\pi$ = $3.1415\ldots$ as the constant $\pi$. With the regression coefficients, $\gamma_{cos}$ and $\gamma_{sine}$ defining the estimated amplitude (vertical extent).

*Model fitting*

We used Stan statistical software (via Rstan package in R statistical software) to approximate the resultant posterior distributions of the parameters using Hamiltonian Monte Carlo algorithms, which is a variant of Markov Chain Monte Carlo (MCMC) methods (4). The value of the discounting variable as well as all covariates except dummy indicators for holiday were mean centered to improve the mixing of MCMC. We generated three independent chains, with each chain containing 3,000 iterations as burn-in sample, followed by 30,000 iterations as the samples from the resultant posterior distribution. For each chain, we inspected the convergence of the MCMC visually and numerically based on the values of the effective number of sample size and Rhat for each model (5). Stan codes to specify the model, and R codes to generate example data to fit the model are available in: https://github.com/hiroshimamiya/promotionLag/.

*Prior specification*

The natural log-transformed sales outcome $Y_{t}$ follows a normal distribution, with standard deviation $\sigma_{\epsilon}$ . We assign a half-Cauchy$\left( 0, 5^{2} \right)$ prior to $\sigma_{\epsilon}$.The half-Cauchy distribution is commonly suggested as a prior for a scale parameter (6). This is a positive half of the Cauchy distribution (constrained to take positive values as scale parameters are always positive). As a sensitivity analysis, we also assigned prior distributions with varying diffuseness (scale of the Cauchy distribution), half-Cauchy$\left( 0, 3^{2} \right)$ and half-Cauchy$\left( 0, {10}^{2} \right)$, which generated nearly identical results to the original analysis, suggesting that the resultant posterior distributions are not sensitive to the prior specification of this scale parameter. We also used a positive-constrained normal distribution to examine posterior sensitivity with the same values of the scaling parameter as above, which led to nearly identical results.

The prior probability of the immediate effect of discounting ($\beta$), the season coefficients ($\gamma_{Cos} and \gamma_{Sine})$, as well as the coefficients of the covariates ($\delta$) were specified as independent non-informative normal distributions $\mathrm{Normal}\left( 0,5^{2} \right)$. The use of alternative prior specifications, Normal$\left( 0,3^{2} \right)$ and Normal$\left( 0,{10}^{2} \right)$,did not result in noticeably different posterior distributions.

For the time-varying intercept, $\alpha_{t}$, the prior probability of the initial value at $t = 1$ was $\alpha_{1}\sim\mathrm{Normal}\left( 0,5^{2} \right)$, which was subsequently allowed to evolve as $\alpha_{t}\sim\mathrm{Normal}\left( \alpha_{t-1},\epsilon_{alpha_{t}} \right)$ with a random noise $\epsilon_{alpha_{t}} \sim$ half-Normal(0,1). As another sensitivity analysis, we also assigned a smoother transition (i.e., smaller fluctuation over time) by assigning $\alpha_{t}$ ~ half-Normal$(0, {0.1}^{2})$ and $\alpha_{t}$ ~ half-Normal$(0, {0.5}^{2})$, with nearly identical results. The lag parameter was assigned a non-informative uniform distribution $\lambda\sim Uniform\left( 0,1 \right)$, which ensures the monotonic decay of the association towards zero as stated above.


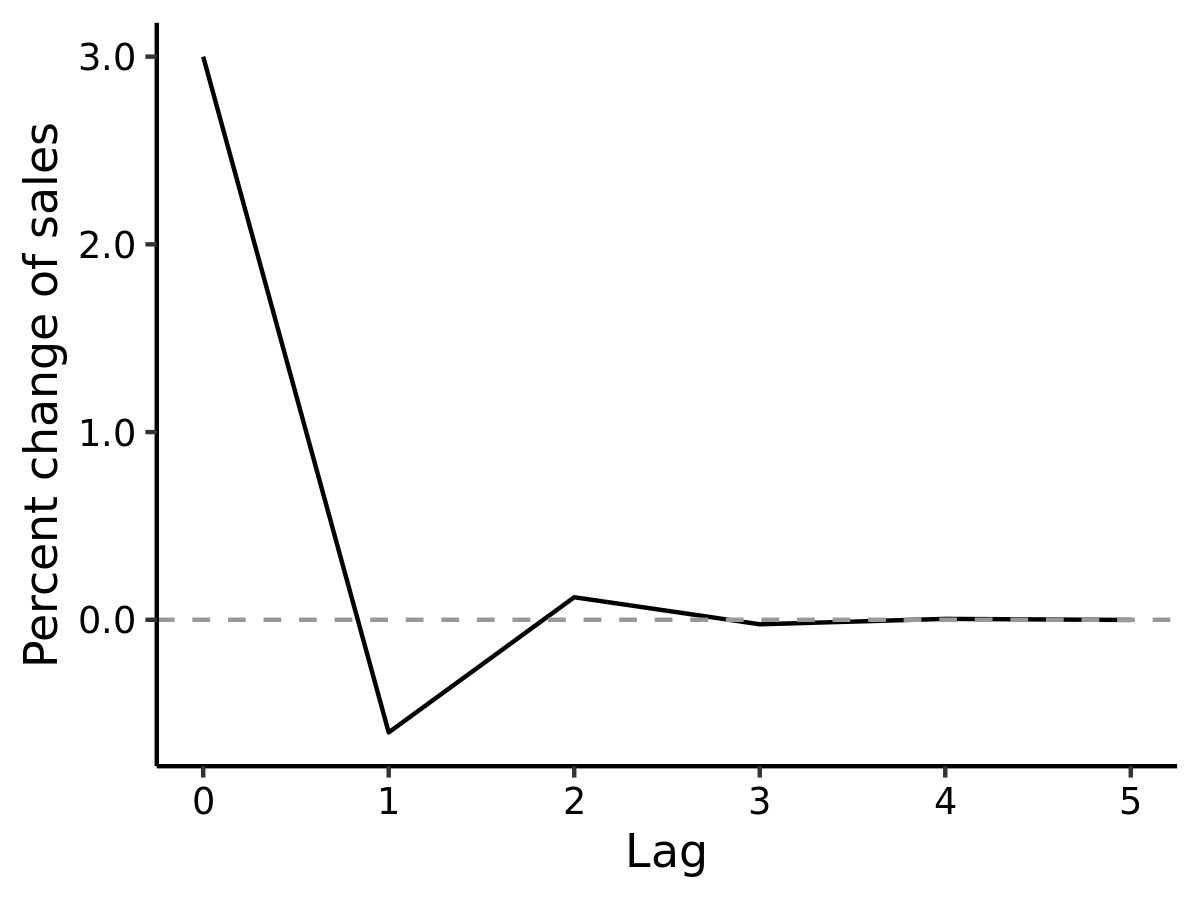


Supplementary Figure S3. Post-promotion dip of sales at one week after the discounting period (*x* = 0).

The y-axis represents the percent change of sales relative to the baseline (pre-discounting) sales due to one percent discounting. Unlike the monotonic decay seen in Supplementary Figure S2 a and b, the oscillating pattern described here is captured by constraining the value of $\lambda$ as: $-1 <\lambda<0$. The decay function in this plot is generated by $\lambda=-0.2.$The dip is attributed to reduced purchasing activities if households stockpile the promoted items during the discounting period. However, the dip may not be observed when stockpiled items are consumed rapidly, leading to an immediate re-purchasing after discounting (7–9).


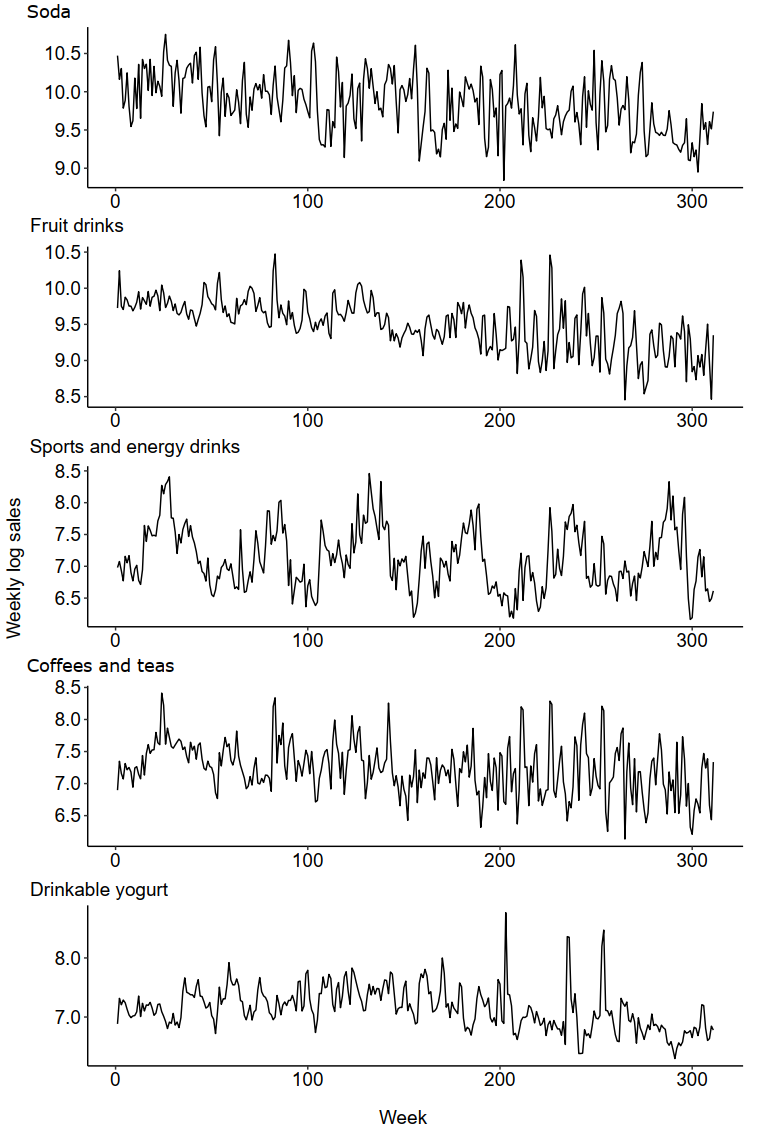


Supplementary Figure S4: Weekly series of category-level percent sales.


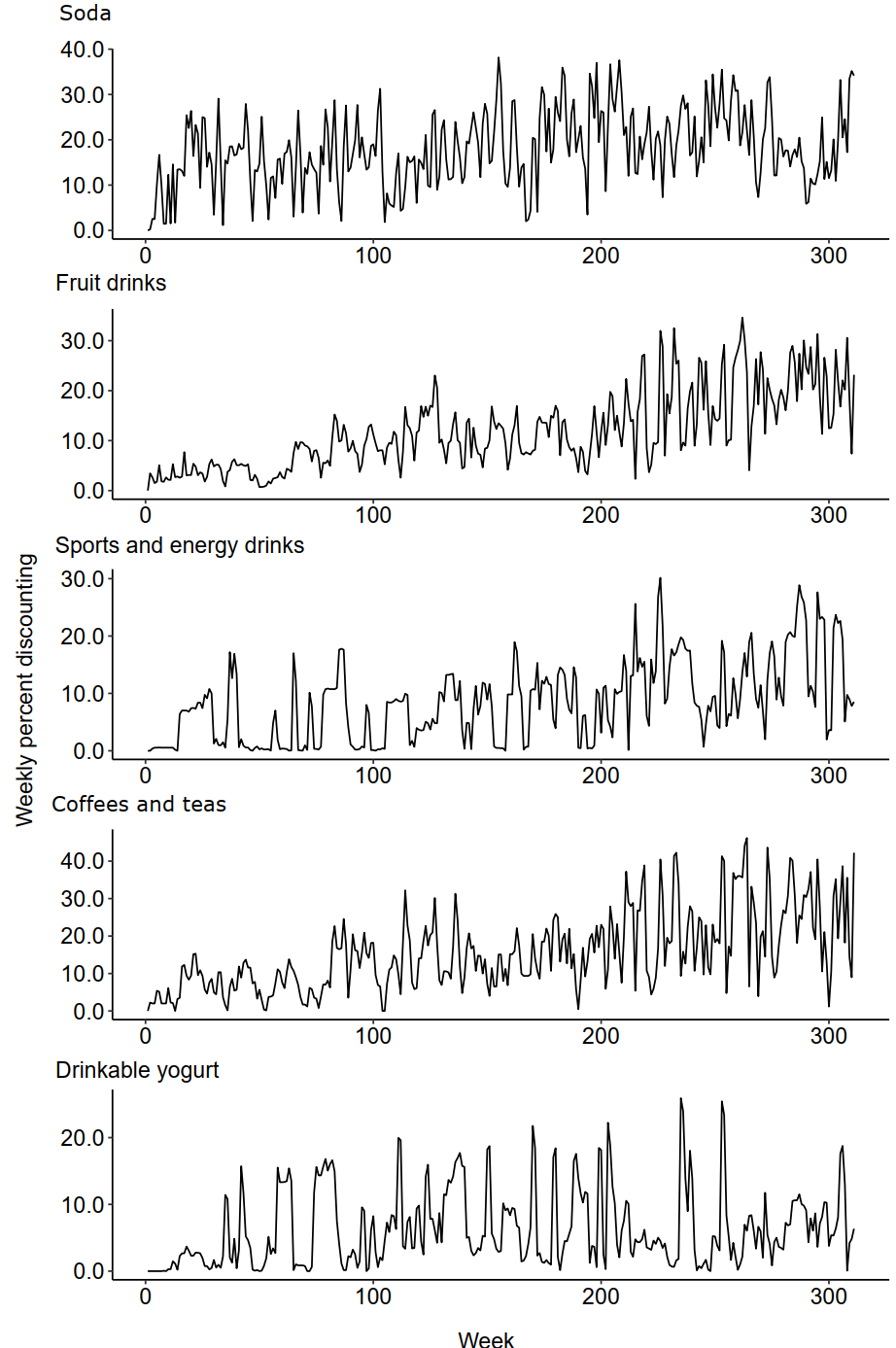


Supplementary Figure S5: Weekly series of category-level percent discounting.


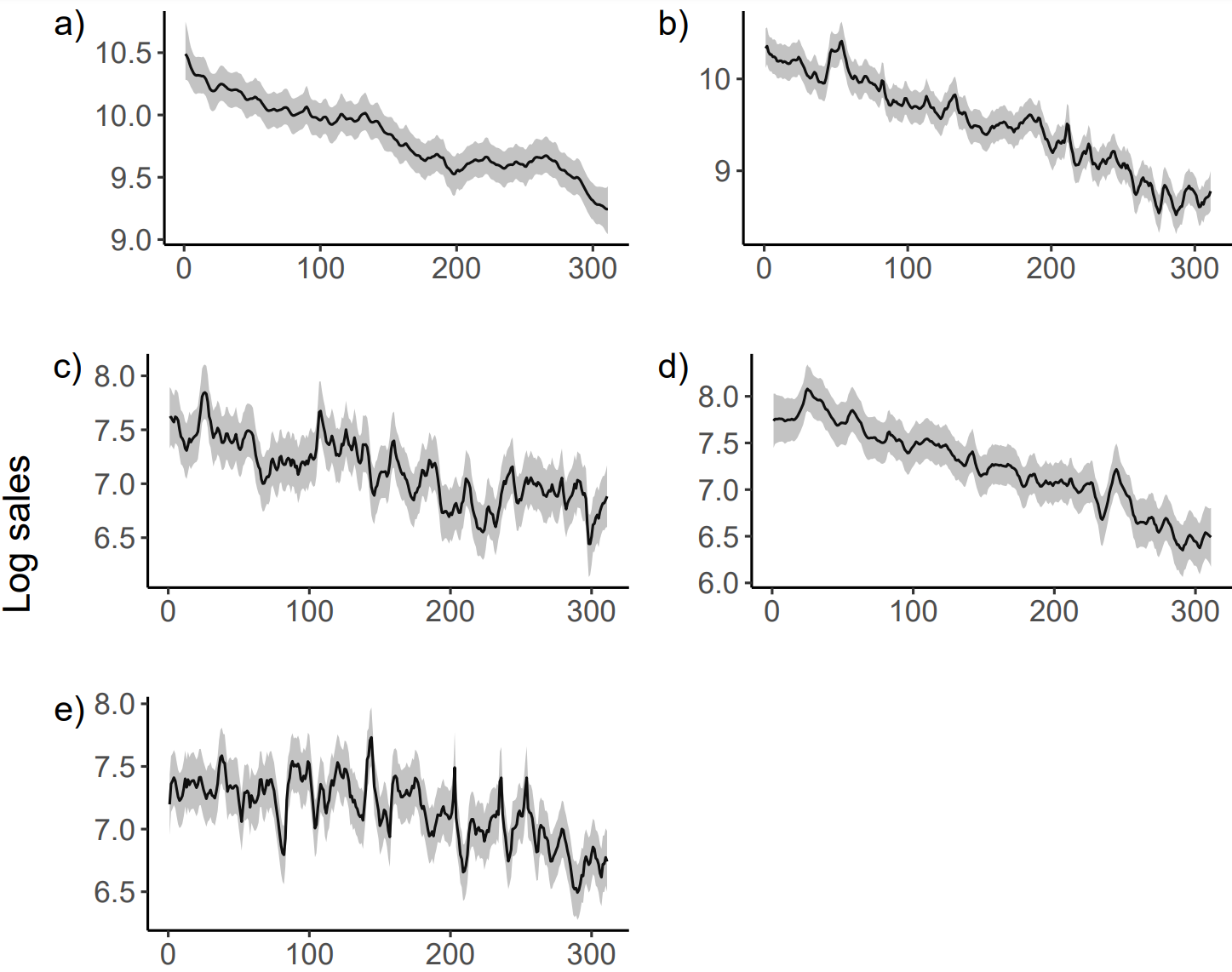


Supplementary Figure S6. Posterior mean (solid line) and 95% credible interval (grey shaded area) of weekly-varying intercept, $\alpha_{t}$, of log sales for a) soda, b) fruit drinks, c) sports and energy drinks, d) coffees and teas, and e) drinkable yogurts. Note that the value of the discounting variable was mean centered; therefore, the estimated level of the intercept represents the sales at the mean level of discounting.


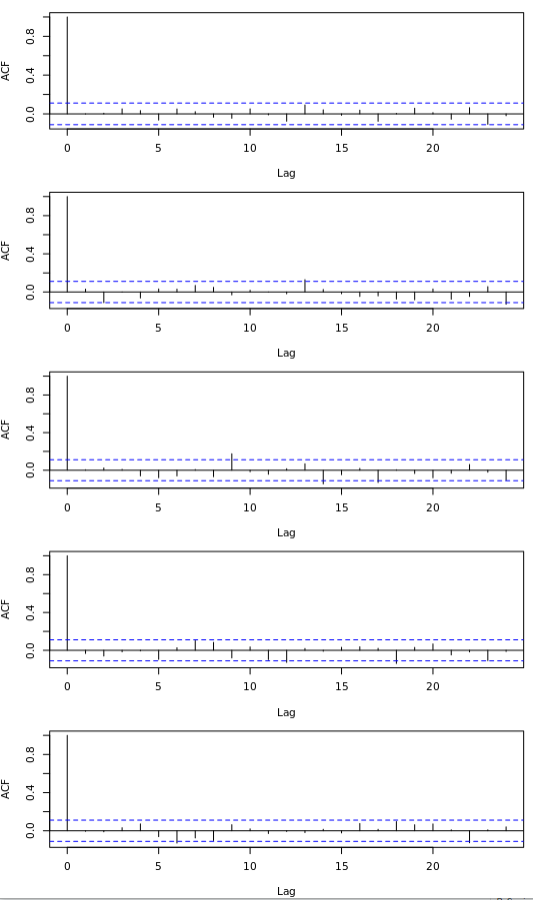


Supplementary Figure S7**.** Autocorrelation function of residuals for : soda, fruit drinks, sports and energy drinks, sweetened coffees and teas, and sweetened drinkable yogurt category, ordered from the top. The grey band around solid line indicates 95% credible interval. It should be noted that the fitted sales in these plots do not represent a forecasting distribution.

Supplementary Table S1. Covariates retained in each beverage model.

| **Beverage category** | **Covariates** |
| --- | --- |
| Soda | Holiday |
| Fruit juice | Holiday, Display |
| Sport and energy drinks | Holiday, Display |
| Sugar-sweetened coffee and tea | Holiday, Price |
| Sugar-sweetened drinkable yogurt | Holiday, Display |

Holiday is an indicator variable for week containing provincial and national statutory holidays.

Display is the weekly weighted proportion of individual beverage items promoted at prominent locations in the store, where the weights represent the normalized sales quantity of individual beverage items within each category. Price is the weekly regular (non-discount) price of each category calculated as the weighted mean of individual beverage items.

Supplementary Table S2. Non-log serving-standardized sales quantity of SSB categories attributed to lagged effect along (left pane) and both lagged and immediate effect (right pane).

|  | Sales due to lagged effect | | |  | Sales due to lagged and immediate effect | | |
| --- | --- | --- | --- | --- | --- | --- | --- |
| SSB category | Mean | Median | 95%CI |  | Mean | Median | 95%CI |
| Soda | 306,483 | 238,042 | (9,943- 977,152) |  | 4,308,369 | 4,283,077 | (3,418,117 - 5,343,486) |
|  |  |  |  |  |  |  |  |
| Fruits drinks | 195,659 | 174,491 | (10,659 - 508,513) |  | 2,317,649 | 2,306,233 | (1,876,257 - 2,824,259) |
|  |  |  |  |  |  |  |  |
| Energy and sports drinks | 28,459 | 26,345 | (2,661 - 67,253) |  | 131,606 | 130,446 | (96,155 - 173,625) |
|  |  |  |  |  |  |  |  |
| Sweetened coffees and teas | 20,079 | 16,627 | (802 - 58,650 |  | 281,883 | 279,539 | (216,303 - 360,958) |
|  |  |  |  |  |  |  |  |
| Sweetened drinkable yogurts | 6,007 | 4,870 | (213- 18,060 |  | 133,117 | 132,531 | (108,460 - 161,073) |


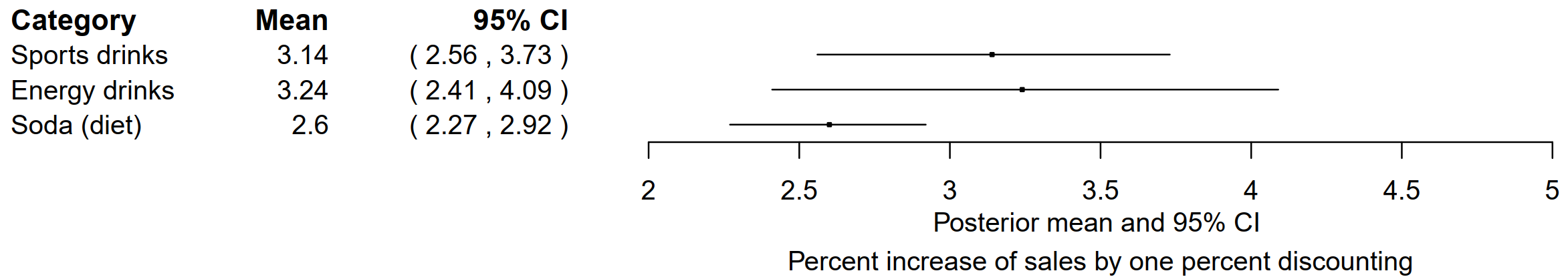


**Supplementary Figure S8.** The estimated immediate effect $\beta$ for price discounting on the sales of sports drinks, energy drinks, and diet soda. The value represents the percent increase of non-log sales upon one percent discounting for each SSB category, as calculated by multiplying the posterior summary of $\beta$ by 100.


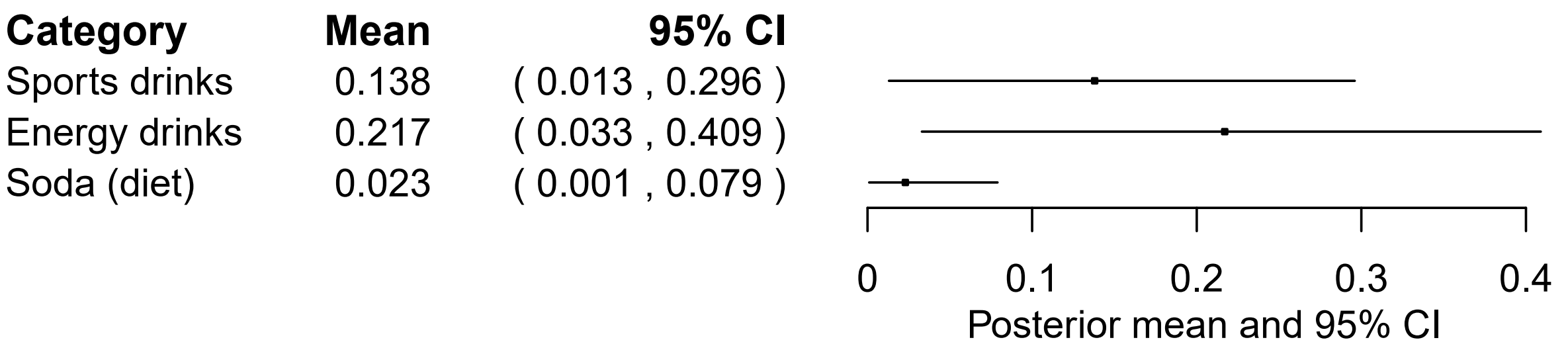


Supplementary Figure S9. Posterior summary of the estimated lag coefficient, $\lambda$, for price discounting on the sales of sports drinks, energy drinks, and diet soda. The variable $\lambda$ represents a unitless quantity, whose value ranges from 0 to 1, with 0 representing the absence of lag.


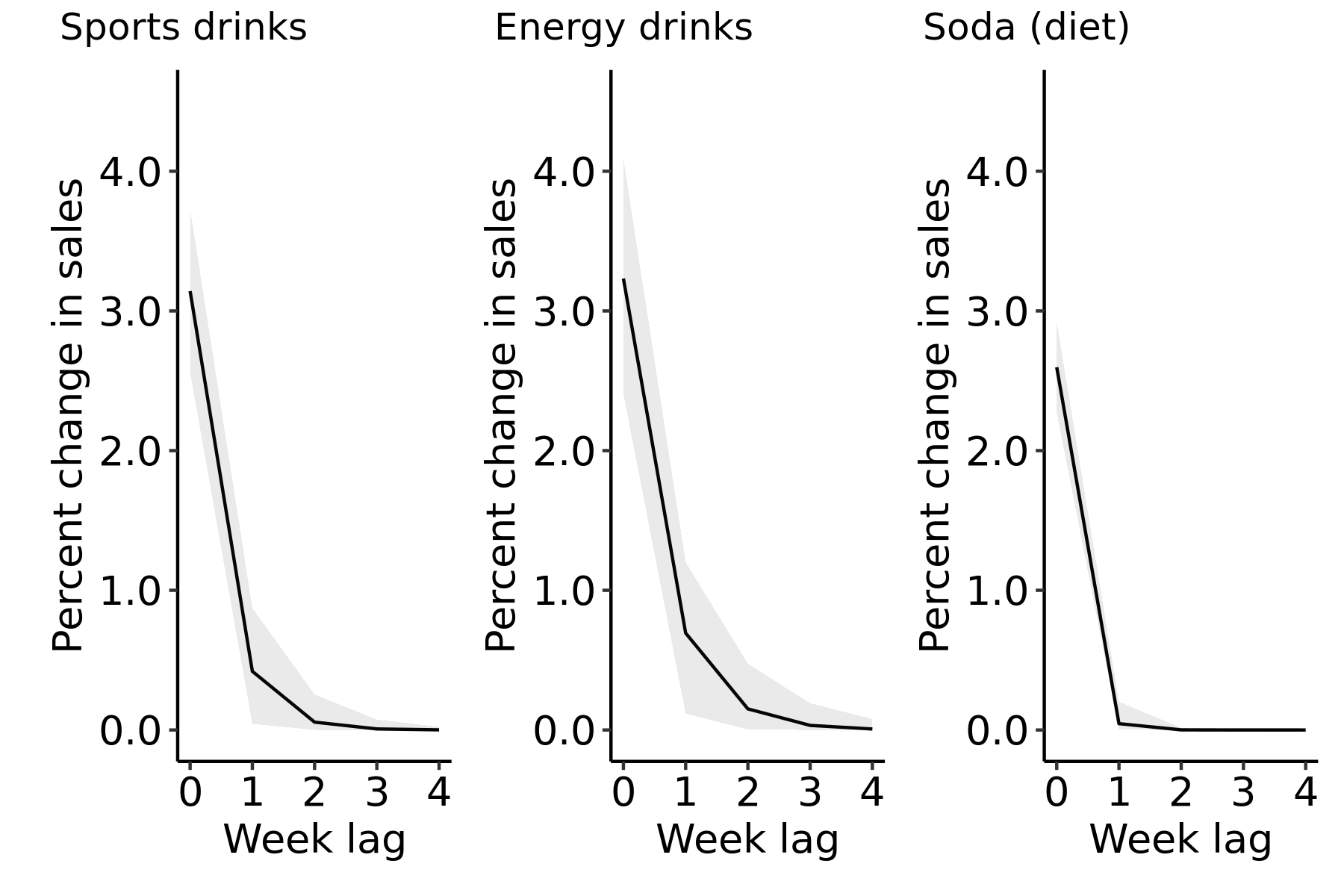


Supplementary Figure S10. Impulse response function showing the lagged effect of price discounting on the sales of sports drinks, energy drinks, and diet soda. The gray shaded area indicates 95% Credible Interval. The value at *x* = 0 represents the immediate effect represented by the posterior median of $\beta$, the percent change of sales during the period of one percent discounting

# References for Supplementary Information File

1. West M, Harrison J. Bayesian forecasting and dynamic models. Second edition. New York: Springer; 1997. (Springer series in statistics).

2. Ravines RR, Schmidt AM, Migon HS. Revisiting distributed lag models through a Bayesian perspective. Appl Stoch Models Bus Ind. 2006;22(2):193–210.

3. Petris G, Petrone S, Campagnoli P. Dynamic linear models with R. New York: Springer-Verlag; 2009. (Use R!).

4. Carpenter B, Gelman A, Hoffman MD, Lee D, Ben Goodrich, Betancourt M, et al. Stan: a probabilistic programming language. Journal of Statistical Software. 2017 Jan 11;76(1):1–32.

5. Brooks SP, Gelman A. General Methods for Monitoring Convergence of Iterative Simulations. Journal of Computational and Graphical Statistics. 1998;7(4):434–55.

6. Gelman A. Prior distributions for variance parameters in hierarchical models. Bayesian Analysis. 2006;1(3):515–33.

7. Hendel I, Nevo A. The post-promotion dip puzzle: what do the data have to say? Quantitative Marketing and Economics. 2003 Dec 1;1(4):409–24.

8. van Heerde HJ, Neslin SA. Sales promotion models. In: Wierenga B, van der Lans R, editors. Handbook of marketing decision models. Springer International Publishing; 2017. p. 13–77. (International Series in Operations Research & Management Science).

9. Neslin SA, Schneider Stone LG. Consumer inventory sensitivity and the postpromotion dip. Market Lett. 1996 Jan 1;7(1):77–94.
